# (1*S*,3*R*,8*R*,11*S*)-11-Bromo-10-bromo­methyl-2,2-di­chloro-3,7,7-tri­methyl­tricyclo­[6.4.0.0^1,3^]dodec-9-ene

**DOI:** 10.1107/S1600536813019697

**Published:** 2013-07-20

**Authors:** Ahmed Benharref, Jamal El karroumi, Lahcen El Ammari, Mohamed Saadi, Moha Berraho

**Affiliations:** a Laboratoire de Chimie des Substances Naturelles, "Unité Associé au CNRST (URAC16)", Faculté des Sciences Semlalia, BP 2390 Bd My Abdellah, 40000 Marrakech, Morocco; b Laboratoire de Chimie du Solide, Appliquée, Faculté des Sciences, Université MohammedV-Agdal , Avenue Ibn Battouta, BP 1014, Rabat, Morocco; c Laboratoire de Chimie du Solide, Appliquée, Faculté des Sciences, Université MohammedV-Agdal, Avenue Ibn Battouta, BP 1014, Rabat, Morocco

## Abstract

The title compound, C_16_H_22_Br_2_Cl_2_, was synthesized from β-him­achalene (3,5,5,9-tetra­methyl-2,4a,5,6,7,8-hexa­hydro-1*H*-benzo­cyclo­heptene), which was isolated from the essential oil of the Atlas cedar (*Cedrus Atlantica*). The mol­ecule is built up from fused six- and seven-membered rings and an appended three-membered ring. The six-membered ring has a half-chair conformation, whereas the seven-membered ring displays a chair conformation. The dihedral angle between the two best plane through each ring is 59.5 (2)°. No specific inter­molecular inter­actions were discerned in the crystal packing.

## Related literature

For the reactivity and biological properties of β-himachalene, see: El Haib *et al.* (2011 ▶); El Jamili *et al.* (2002 ▶); Daoubi *et al.* (2004 ▶). For related structures, see: Oukhrib *et al.* (2013 ▶); Ourhriss *et al.* (2013 ▶); Benharref *et al.*(2013 ▶). For puckering parameters, see: Cremer & Pople (1975 ▶).
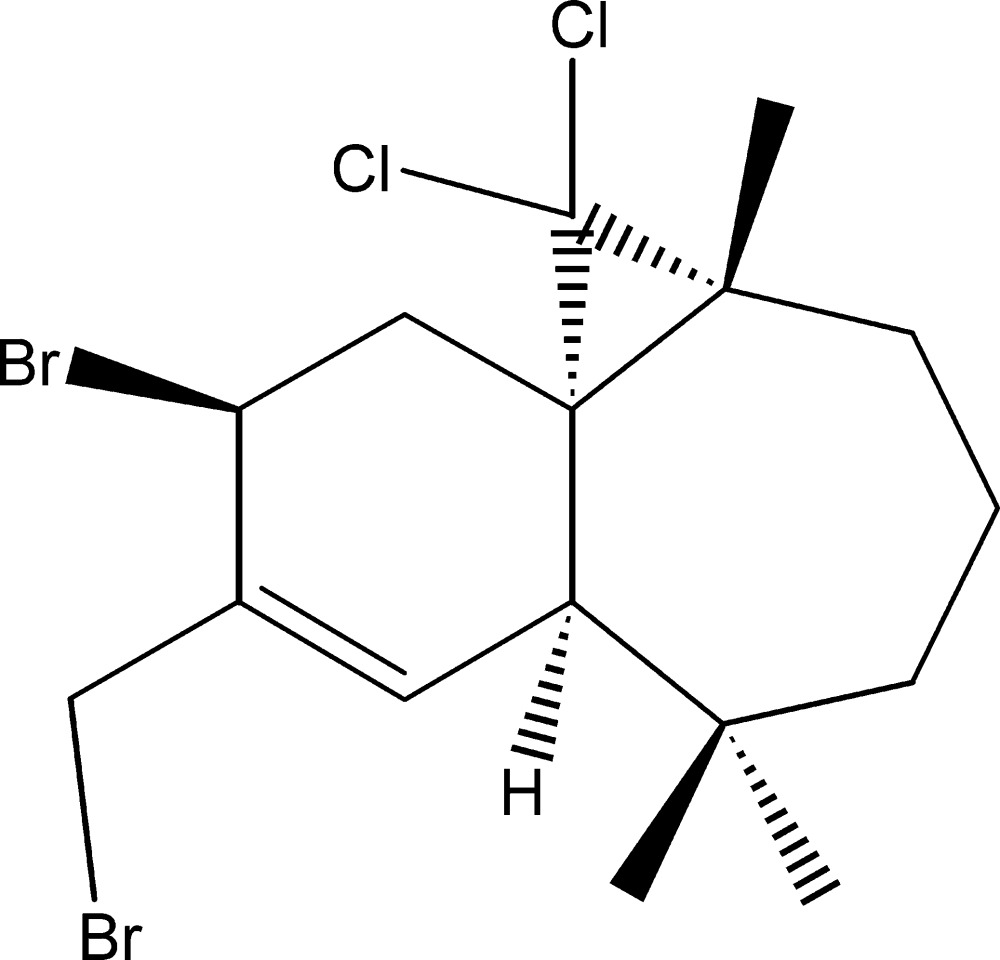


## Experimental

### 

#### Crystal data

C_16_H_22_Br_2_Cl_2_
*M* *_r_* = 445.06
Orthorhombic, 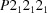

*a* = 8.2594 (7) Å
*b* = 13.0352 (11) Å
*c* = 16.6241 (13) Å
*V* = 1789.8 (3) Å^3^
*Z* = 4
Mo *K*α radiation
μ = 4.82 mm^−1^
*T* = 293 K
0.20 × 0.15 × 0.12 mm

#### Data collection

Bruker APEXII CCD diffractometer
Absorption correction: multi-scan (*SHELXS97*; Sheldrick,2008 ▶) *T*_min_ = 0.423, *T*_max_ = 0.617
10693 measured reflections
3654 independent reflections
3183 reflections with *I* > 2σ(*I*)
*R*_int_ = 0.026

#### Refinement

*R*[*F* ^2^ > 2σ(*F* ^2^)] = 0.038
*wR*(*F* ^2^) = 0.102
*S* = 1.05
3654 reflections
184 parameters
H-atom parameters constrained
Δρ_max_ = 0.55 e Å^−3^
Δρ_min_ = −0.54 e Å^−3^
Absolute structure: Flack & Bernardinelli (2000 ▶), 614 Friedel pairs
Absolute structure parameter: 0.022 (13)

### 

Data collection: *APEX2* (Bruker, 2009 ▶); cell refinement: *SAINT* (Bruker, 2009 ▶); data reduction: *SAINT*; program(s) used to solve structure: *SHELXS97* (Sheldrick, 2008 ▶); program(s) used to refine structure: *SHELXL97* (Sheldrick, 2008 ▶); molecular graphics: *ORTEP-3 for Windows* (Farrugia, 2012 ▶); software used to prepare material for publication: *WinGX* (Farrugia, 2012 ▶).

## Supplementary Material

Crystal structure: contains datablock(s) I, global. DOI: 10.1107/S1600536813019697/tk5241sup1.cif

Structure factors: contains datablock(s) I. DOI: 10.1107/S1600536813019697/tk5241Isup2.hkl

Supplementary material file. DOI: 10.1107/S1600536813019697/tk5241Isup3.cml

Additional supplementary materials:  crystallographic information; 3D view; checkCIF report

## Figures and Tables

**Fig. 1.**
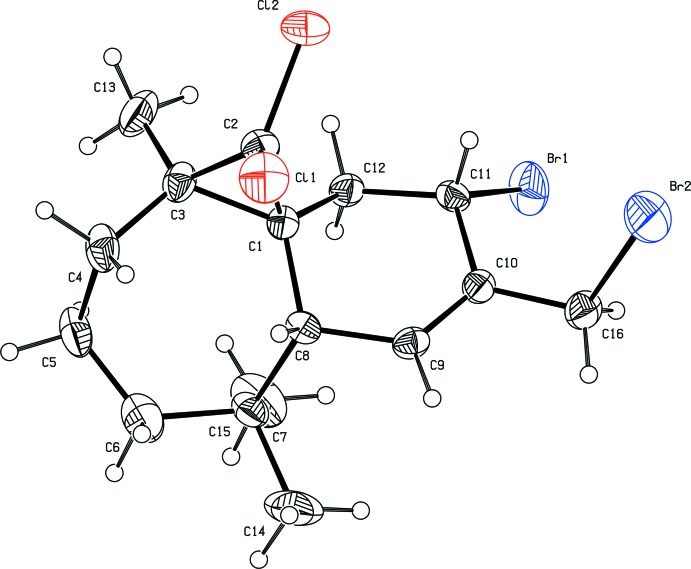
Molecular structure of the title compound with the atom-labelling scheme. Displacement ellipsoids are drawn at the 30% probability. level. H atoms are represented as small spheres of arbitrary radii.

## supplementary crystallographic information

### Comment

The bicyclic sesquiterpene β-himachalene is the main constituent (50%) of the essential oil of the Atlas cedar (*Cedrus atlantica*) (El Haib *et al.*, 2011). The reactivity of this sesquiterpene and its derivatives has been studied extensively by our team in order to prepare new products having biological proprieties (El Jamili *et al.*, 2002; Daoubi *et al.*, 2004; Ourhriss *et al.*, 2013; Oukhrib *et al.*, 2013; Benharref *et al.*, 2013). In this work, we present the crystal structure of the title compound, (1*S*,3*R*,8*R*,11*S*)- 10- bromomethyl-11-bromo-2,2-dichloro-3,7,7-trimethyltricyclo [6.4.0.0_1_,_3_] dodec-9-ene. The molecule contains fused six-and seven-membered rings, which is fused to a three-membered ring as shown in Fig. 1. The six-membered ring has a half-chair conformation, as indicated by the total puckering amplitude QT = 0.466 (4) Å and spherical polar angle θ = 129.9 (7)° with φ = 152.5 (7)°, whereas the seven-membered ring displays a chair conformation with QT = 0.8129 (51) Å, θ = 32.71 (40)°, φ2 = -46.29 (5)° and φ3 = -77.86 (39)° (Cremer & Pople, 1975). Owing to the presence of Br atoms, the absolute configuration could be fully confirmed, by refining the Flack parameter (Flack & Bernardinelli, 2000) as C1(*S*), C3(*R*), C8(*R*) and C11(*S*).

### Experimental

In a reactor equipped with a stirrer, a condenser, a dropping funnel and a thermometer containing (1*S*,3*R*,8*R*)-2,2-dichloro-3,7,7,10-tetramethyltricyclo [6.4.0.01,3] dodec-9-ene (2 g, 7 mmol) (El Jamili *et al.*, 2002) and carbon tetrachloride (60 ml) was added slowly over an 1.5 h by heating and stirring a solution of bromine (1.6 g, 10 mmol) in carbon tetrachloride (5 ml). Heating and stirring were maintained for 1 h after the addition of bromine. Thereafter the reaction mixture was cooled and concentrated to evaporate the carbon tetrachloride. the residue obtained was chromatographed on silica eluting with hexane, which allowed the isolation of pure (1*S*,3*R*,8*R*,11*S*)-10-bromomethyl-11-bromo-2,2-dichloro-3,7,7-trimethyltricyclo [6.4.0.01,3] dodec-9-ene in a yield of 20% (623 mg, 1.4 mmol). The title compound was recrystallized from its hexane solution.

### Refinement

All H atoms were fixed geometrically and treated as riding with C—H = 0.96 Å (methyl), 0.97 Å (methylene), 0.98 Å (methine) with *U*_iso_(H) = 1.2*U*_eq_(methylene, methine) or *U*_iso_(H) = 1.5*U*_eq_(methyl).

### Crystal data

**Table:**

C_16_H_22_Br_2_Cl_2_	*F*(000) = 888
*M**_r_* = 445.06	*D*_x_ = 1.652 Mg m^−^^3^
Orthorhombic, *P*2_1_2_1_2_1_	Mo *K*α radiation, λ = 0.71073 Å
Hall symbol: P 2ac 2ab	Cell parameters from 3653 reflections
*a* = 8.2594 (7) Å	θ = 2.8–26.4°
*b* = 13.0352 (11) Å	µ = 4.82 mm^−^^1^
*c* = 16.6241 (13) Å	*T* = 293 K
*V* = 1789.8 (3) Å^3^	Block, colourless
*Z* = 4	0.20 × 0.15 × 0.12 mm

### Data collection

**Table:**

Bruker APEXII CCD diffractometer	3654 independent reflections
Radiation source: fine-focus sealed tube	3183 reflections with *I* > 2σ(*I*)
Graphite monochromator	*R*_int_ = 0.026
ω and φ scans	θ_max_ = 26.4°, θ_min_ = 2.5°
Absorption correction: multi-scan *SHELXS97* (Sheldrick,2008)	*h* = −10→10
*T*_min_ = 0.423, *T*_max_ = 0.617	*k* = −13→16
10693 measured reflections	*l* = −11→20

### Refinement

**Table:**

Refinement on *F*^2^	Secondary atom site location: difference Fourier map
Least-squares matrix: full	Hydrogen site location: inferred from neighbouring sites
*R*[*F*^2^ > 2σ(*F*^2^)] = 0.038	H-atom parameters constrained
*wR*(*F*^2^) = 0.102	*w* = 1/[σ^2^(*F*_o_^2^) + (0.0406*P*)^2^ + 1.2463*P*] where *P* = (*F*_o_^2^ + 2*F*_c_^2^)/3
*S* = 1.05	(Δ/σ)_max_ < 0.001
3654 reflections	Δρ_max_ = 0.55 e Å^−^^3^
184 parameters	Δρ_min_ = −0.54 e Å^−^^3^
0 restraints	Absolute structure: Flack & Bernardinelli (2000), 614 Friedel pairs
Primary atom site location: structure-invariant direct methods	Flack parameter: 0.022 (13)

### Special details

**Table:**

Geometry. All s.u.'s (except the s.u. in the dihedral angle between two l.s. planes) are estimated using the full covariance matrix. The cell s.u.'s are taken into account individually in the estimation of s.u.'s in distances, angles and torsion angles; correlations between s.u.'s in cell parameters are only used when they are defined by crystal symmetry. An approximate (isotropic) treatment of cell s.u.'s is used for estimating s.u.'s involving l.s. planes.
Refinement. Refinement of *F*^2^ against ALL reflections. The weighted *R*-factor *wR* and goodness of fit *S* are based on *F*^2^, conventional *R*-factors *R* are based on *F*, with *F* set to zero for negative *F*^2^. The threshold expression of *F*^2^ > 2σ(*F*^2^) is used only for calculating *R*-factors(gt) *etc*. and is not relevant to the choice of reflections for refinement. *R*-factors based on *F*^2^ are statistically about twice as large as those based on *F*, and *R*- factors based on ALL data will be even larger.

### Fractional atomic coordinates and isotropic or equivalent isotropic displacement parameters (Å^2^)

**Table:**

	*x*	*y*	*z*	*U*_iso_*/*U*_eq_	
C1	0.3469 (4)	0.4202 (3)	0.7271 (2)	0.0346 (8)	
C2	0.1795 (4)	0.3776 (4)	0.7462 (3)	0.0440 (9)	
C3	0.2012 (4)	0.4409 (4)	0.6715 (2)	0.0446 (9)	
C4	0.1967 (6)	0.3884 (4)	0.5903 (3)	0.0570 (12)	
H4A	0.0915	0.3999	0.5659	0.068*	
H4B	0.2095	0.3151	0.5979	0.068*	
C5	0.3291 (6)	0.4270 (5)	0.5327 (3)	0.0598 (13)	
H5A	0.2927	0.4188	0.4775	0.072*	
H5B	0.3480	0.4994	0.5421	0.072*	
C6	0.4858 (7)	0.3684 (5)	0.5444 (3)	0.0721 (15)	
H6A	0.5565	0.3853	0.4998	0.086*	
H6B	0.4610	0.2959	0.5401	0.086*	
C7	0.5838 (5)	0.3841 (3)	0.6231 (2)	0.0446 (9)	
C8	0.4798 (5)	0.3470 (3)	0.6986 (2)	0.0354 (8)	
H8	0.4247	0.2840	0.6817	0.043*	
C9	0.5846 (5)	0.3179 (3)	0.7684 (2)	0.0384 (8)	
H9	0.6527	0.2619	0.7611	0.046*	
C10	0.5903 (4)	0.3636 (3)	0.8394 (2)	0.0373 (8)	
C11	0.4874 (5)	0.4553 (3)	0.8571 (2)	0.0370 (8)	
H11	0.4009	0.4346	0.8940	0.044*	
C12	0.4109 (5)	0.5026 (3)	0.7827 (2)	0.0391 (8)	
H12A	0.3228	0.5475	0.7987	0.047*	
H12B	0.4908	0.5436	0.7545	0.047*	
C13	0.1283 (6)	0.5488 (4)	0.6685 (3)	0.0684 (14)	
H13A	0.1301	0.5784	0.7214	0.103*	
H13B	0.0185	0.5448	0.6498	0.103*	
H13C	0.1904	0.5907	0.6325	0.103*	
C14	0.7278 (9)	0.3099 (6)	0.6141 (4)	0.093 (2)	
H14A	0.8067	0.3242	0.6550	0.139*	
H14B	0.7761	0.3188	0.5620	0.139*	
H14C	0.6904	0.2406	0.6196	0.139*	
C15	0.6474 (9)	0.4891 (5)	0.6292 (4)	0.0845 (19)	
H15A	0.5591	0.5365	0.6345	0.127*	
H15B	0.7083	0.5052	0.5816	0.127*	
H15C	0.7165	0.4943	0.6754	0.127*	
C16	0.6964 (5)	0.3222 (4)	0.9042 (3)	0.0507 (10)	
H16A	0.7645	0.3769	0.9247	0.061*	
H16B	0.7665	0.2698	0.8817	0.061*	
Cl3	0.14150 (16)	0.24471 (10)	0.74083 (8)	0.0694 (3)	
Cl4	0.06998 (15)	0.42878 (14)	0.82812 (8)	0.0734 (4)	
Br1	0.61787 (8)	0.56321 (4)	0.90951 (3)	0.07068 (18)	
Br2	0.57150 (8)	0.26329 (6)	0.99305 (4)	0.0885 (2)	

### Atomic displacement parameters (Å^2^)

**Table:**

	*U*^11^	*U*^22^	*U*^33^	*U*^12^	*U*^13^	*U*^23^
C1	0.0338 (17)	0.035 (2)	0.0352 (18)	−0.0032 (15)	−0.0007 (13)	0.0027 (15)
C2	0.0327 (18)	0.053 (3)	0.046 (2)	−0.0103 (17)	0.0056 (15)	0.0024 (19)
C3	0.0342 (18)	0.051 (2)	0.048 (2)	−0.0075 (18)	−0.0073 (15)	0.010 (2)
C4	0.051 (2)	0.076 (3)	0.045 (2)	−0.021 (2)	−0.0154 (19)	0.004 (2)
C5	0.066 (3)	0.080 (3)	0.033 (2)	−0.020 (3)	−0.0090 (18)	0.011 (2)
C6	0.075 (3)	0.091 (4)	0.051 (3)	−0.017 (3)	0.001 (2)	−0.001 (3)
C7	0.045 (2)	0.055 (2)	0.0338 (19)	−0.007 (2)	0.0081 (16)	−0.0015 (17)
C8	0.0395 (19)	0.033 (2)	0.0341 (19)	−0.0040 (15)	0.0043 (14)	−0.0020 (15)
C9	0.0385 (19)	0.0315 (19)	0.045 (2)	0.0062 (15)	0.0033 (16)	0.0031 (16)
C10	0.0346 (18)	0.040 (2)	0.0373 (19)	−0.0006 (16)	0.0011 (15)	0.0059 (16)
C11	0.0401 (19)	0.037 (2)	0.0336 (19)	0.0006 (15)	0.0026 (14)	−0.0077 (16)
C12	0.040 (2)	0.036 (2)	0.041 (2)	0.0055 (16)	−0.0017 (16)	−0.0041 (16)
C13	0.052 (3)	0.075 (4)	0.079 (3)	0.019 (3)	−0.015 (2)	0.016 (3)
C14	0.085 (4)	0.112 (5)	0.082 (4)	0.024 (4)	0.046 (4)	0.006 (4)
C15	0.097 (5)	0.082 (4)	0.074 (4)	−0.046 (4)	0.021 (3)	−0.004 (3)
C16	0.046 (2)	0.057 (3)	0.049 (2)	0.0047 (18)	0.0030 (19)	0.017 (2)
Cl3	0.0657 (7)	0.0632 (8)	0.0793 (8)	−0.0286 (6)	0.0057 (6)	0.0147 (6)
Cl4	0.0472 (6)	0.1082 (11)	0.0649 (7)	0.0060 (7)	0.0215 (5)	0.0002 (8)
Br1	0.0901 (4)	0.0583 (3)	0.0637 (3)	−0.0039 (3)	−0.0277 (3)	−0.0181 (2)
Br2	0.0869 (4)	0.1127 (5)	0.0659 (4)	−0.0004 (4)	0.0151 (3)	0.0420 (3)

### Geometric parameters (Å, º)

**Table:**

C1—C12	1.513 (5)	C8—H8	0.9800
C1—C2	1.524 (5)	C9—C10	1.323 (6)
C1—C8	1.529 (5)	C9—H9	0.9300
C1—C3	1.541 (5)	C10—C16	1.489 (6)
C2—C3	1.502 (6)	C10—C11	1.495 (5)
C2—Cl3	1.763 (5)	C11—C12	1.520 (5)
C2—Cl4	1.765 (4)	C11—Br1	1.975 (4)
C3—C4	1.514 (7)	C11—H11	0.9800
C3—C13	1.531 (7)	C12—H12A	0.9700
C4—C5	1.538 (6)	C12—H12B	0.9700
C4—H4A	0.9700	C13—H13A	0.9600
C4—H4B	0.9700	C13—H13B	0.9600
C5—C6	1.515 (8)	C13—H13C	0.9600
C5—H5A	0.9700	C14—H14A	0.9600
C5—H5B	0.9700	C14—H14B	0.9600
C6—C7	1.552 (7)	C14—H14C	0.9600
C6—H6A	0.9700	C15—H15A	0.9600
C6—H6B	0.9700	C15—H15B	0.9600
C7—C15	1.470 (7)	C15—H15C	0.9600
C7—C14	1.540 (8)	C16—Br2	1.959 (4)
C7—C8	1.596 (5)	C16—H16A	0.9700
C8—C9	1.497 (5)	C16—H16B	0.9700
			
C12—C1—C2	116.6 (3)	C9—C8—H8	106.2
C12—C1—C8	112.4 (3)	C1—C8—H8	106.2
C2—C1—C8	119.2 (3)	C7—C8—H8	106.2
C12—C1—C3	121.0 (3)	C10—C9—C8	126.8 (3)
C2—C1—C3	58.7 (3)	C10—C9—H9	116.6
C8—C1—C3	118.9 (3)	C8—C9—H9	116.6
C3—C2—C1	61.2 (2)	C9—C10—C16	120.2 (4)
C3—C2—Cl3	121.2 (3)	C9—C10—C11	121.0 (3)
C1—C2—Cl3	120.6 (3)	C16—C10—C11	118.8 (3)
C3—C2—Cl4	119.5 (3)	C10—C11—C12	113.6 (3)
C1—C2—Cl4	119.2 (3)	C10—C11—Br1	110.2 (3)
Cl3—C2—Cl4	108.6 (2)	C12—C11—Br1	107.3 (3)
C2—C3—C4	119.1 (4)	C10—C11—H11	108.5
C2—C3—C13	118.9 (4)	C12—C11—H11	108.5
C4—C3—C13	112.1 (4)	Br1—C11—H11	108.5
C2—C3—C1	60.1 (2)	C1—C12—C11	110.7 (3)
C4—C3—C1	118.4 (4)	C1—C12—H12A	109.5
C13—C3—C1	119.2 (4)	C11—C12—H12A	109.5
C3—C4—C5	112.9 (4)	C1—C12—H12B	109.5
C3—C4—H4A	109.0	C11—C12—H12B	109.5
C5—C4—H4A	109.0	H12A—C12—H12B	108.1
C3—C4—H4B	109.0	C3—C13—H13A	109.5
C5—C4—H4B	109.0	C3—C13—H13B	109.5
H4A—C4—H4B	107.8	H13A—C13—H13B	109.5
C6—C5—C4	111.3 (4)	C3—C13—H13C	109.5
C6—C5—H5A	109.4	H13A—C13—H13C	109.5
C4—C5—H5A	109.4	H13B—C13—H13C	109.5
C6—C5—H5B	109.4	C7—C14—H14A	109.5
C4—C5—H5B	109.4	C7—C14—H14B	109.5
H5A—C5—H5B	108.0	H14A—C14—H14B	109.5
C5—C6—C7	119.2 (5)	C7—C14—H14C	109.5
C5—C6—H6A	107.5	H14A—C14—H14C	109.5
C7—C6—H6A	107.5	H14B—C14—H14C	109.5
C5—C6—H6B	107.5	C7—C15—H15A	109.5
C7—C6—H6B	107.5	C7—C15—H15B	109.5
H6A—C6—H6B	107.0	H15A—C15—H15B	109.5
C15—C7—C14	108.4 (5)	C7—C15—H15C	109.5
C15—C7—C6	111.6 (5)	H15A—C15—H15C	109.5
C14—C7—C6	103.8 (4)	H15B—C15—H15C	109.5
C15—C7—C8	114.9 (4)	C10—C16—Br2	112.2 (3)
C14—C7—C8	107.6 (4)	C10—C16—H16A	109.2
C6—C7—C8	110.0 (4)	Br2—C16—H16A	109.2
C9—C8—C1	109.4 (3)	C10—C16—H16B	109.2
C9—C8—C7	112.0 (3)	Br2—C16—H16B	109.2
C1—C8—C7	116.2 (3)	H16A—C16—H16B	107.9
